# Burden and Impact of Reactogenicity among Adults Receiving COVID-19 Vaccines in the United States and Canada: Results from a Prospective Observational Study

**DOI:** 10.3390/vaccines12010083

**Published:** 2024-01-13

**Authors:** Matthew D. Rousculp, Kelly Hollis, Ryan Ziemiecki, Dawn Odom, Anthony M. Marchese, Mitra Montazeri, Shardul Odak, Laurin Jackson, Angela Miller, Seth Toback

**Affiliations:** 1 Novavax, Inc., Gaithersburg, MD 20878, USA; amarchese@novavax.com (A.M.M.); mmontazeri@novavax.com (M.M.); amiller@novavax.com (A.M.); stoback@novavax.com (S.T.); 2 RTI Health Solutions, Research Triangle Park, NC 27709, USA; khollis@rti.org (K.H.); rziemiecki@rti.org (R.Z.); dodom@rti.org (D.O.); sodak@rti.org (S.O.); gibson@rti.org (L.J.)

**Keywords:** COVID-19, work impairment, reactogenicity, real-world evidence, absenteeism, presenteeism, SARS-CoV-2, NVX-CoV2373, BNT162b2, mRNA-1273

## Abstract

As SARS-CoV-2 variants continue to emerge, vaccination remains a critical tool to reduce the COVID-19 burden. Vaccine reactogenicity and the impact on work productivity/daily activities are recognized as contributing factors to vaccine hesitancy. To encourage continued COVID-19 vaccination, a more complete understanding of the differences in reactogenicity and impairment due to vaccine-related side effects across currently available vaccines is necessary. The 2019nCoV-406 study (n = 1367) was a prospective observational study of reactogenicity and associated impairments in adults in the United States and Canada who received an approved/authorized COVID-19 vaccine. Compared with recipients of mRNA COVID-19 booster vaccines, a smaller percentage of NVX-CoV2373 booster recipients reported local and systemic reactogenicity. This study’s primary endpoint (percentage of participants with ≥50% overall work impairment on ≥1 of the 6 days post-vaccination period) did not show significant differences. However, the data suggest that NVX-CoV2373 booster recipients trended toward being less impaired overall than recipients of an mRNA booster; further research is needed to confirm this observed trend. The results of this real-world study suggest that NVX-CoV2373 may be a beneficial vaccine option with limited impact on non-work activities, in part due to the few reactogenicity events after vaccination.

## 1. Introduction

COVID-19 is caused by infection with SARS-CoV-2 and continues to be a global burden, with new variants emerging [1]. Several types of vaccines have been developed globally against SARS-CoV-2, many of which are now authorized or approved by regulatory agencies. During 2022, in the United States (US), authorized or approved vaccines included Pfizer-BioNTech’s messenger ribonucleic acid (mRNA) vaccine Comirnaty^®^ (BNT162b2; approved) [2]. Moderna’s mRNA vaccine Spikevax™ (mRNA-1273; authorized) [3] Johnson & Johnson/Janssen’s (J&J) adenovirus vector vaccine (Ad26.COV2.S; no longer available in the US as of June 2023) [4]; and Novavax’s protein-based vaccine (NVX CoV2373; authorized) [5]. In 2022, the same four vaccines were approved in Canada, in addition to AstraZeneca’s adenovirus vector vaccine Vaxzevria™ (ChAdOx1-S [recombinant], formerly AZD1222; approved) and Medicago’s plant-based vaccine Covifenz^®^ (CoVLP; authorized; authorization canceled as of March 2023) [6,7]. The primary series of all authorized/approved COVID-19 vaccines consists of two doses administered 21 or 28 days apart (except for Ad26.COV2.S, which was one dose). Additional booster doses of COVID-19 vaccines are recommended for some populations, including immunocompromised or older individuals [2,3,4,5,6,8].

Emerging variants have exhibited immune evasion, necessitating updates to vaccine strain compositions [9]. In August 2022, the US Food and Drug Administration (FDA) authorized the use of bivalent formulations (based on ancestral and variant strains) of the mRNA vaccines as a single booster dose [10]. Recently, the World Health Organization (WHO), European Medicines Agency (EMA), and US FDA recommended a vaccine strain/composition change to monovalent Omicron XBB.1.5–based vaccines for 2023–2024, for use as a booster dose or for primary series vaccination in those who have not yet been vaccinated against SARS-CoV-2 [11,12,13].

Although the recommended COVID-19 vaccines are effective and have acceptable safety profiles, reactogenic events are seen in many patients. Local or systemic reactogenic events after vaccination include pain, erythema, swelling, lymphadenopathy, fever, headache, myalgia, arthralgia, nausea, vomiting, chills, fatigue, malaise, and use of antipyretic medication [14,15,16]. Reactogenicity may increase after subsequent COVID-19 vaccine doses compared to the first dose [14,15,17]. Reactogenic events for currently authorized/approved COVID-19 vaccines are most commonly mild and transient, resolving in most recipients within 1–2 days [14,15,18,19,20], but may impact the recipient’s ability to perform daily tasks, leading to absenteeism (any time off from work) or presenteeism (impaired performance at work).

Information regarding the impact of COVID-19 vaccine side effects on work absences/impairments is limited. Improved understanding of the comparative risk-benefit profiles (especially regarding reactogenicity) is critical as additional COVID-19 vaccine options and doses become available and the world considers transitioning to annual vaccination. Lower levels of reactogenicity will likely help to optimize COVID-19 vaccine uptake and reduce vaccine hesitancy [21,22,23,24,25,26,27,28].

The 2019nCoV-406 study examined the impact of common reactogenic events from the COVID-19 vaccination on absenteeism, presenteeism, and work productivity loss. These factors were compared for adults in the US and Canada receiving NVX-CoV2373 versus other authorized/approved COVID-19 vaccines in a real-world setting. The study used several questionnaires to solicit responses, including the Absenteeism and Presenteeism Diary, the Vaccine Symptoms Diary, and the Vaccine Healthcare Resource Utilization (HCRU) survey.

## 2. Materials and Methods

### 2.1. Study Design

**Figure 1:**
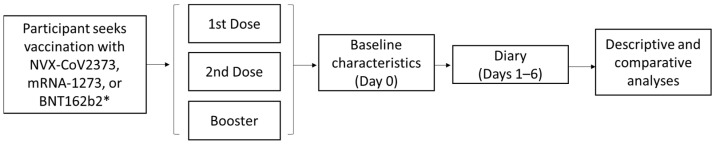
Study design for the 2019nCoV-406 study. * Participants who received vaccines other than BNT162b2, mRNA-1273, or NVX-CoV2373 were excluded from analyses due to low numbers.

The 2019nCoV-406 study was a prospective, non-interventional, observational study of working adults in the US and Canada who received an approved/authorized COVID-19 vaccine dose. Participants completed a baseline survey (Figure S1) on the day they received a vaccine and a daily diary for 6 days after vaccine receipt (Absenteeism and Presenteeism Diary and Vaccine Symptoms Diary). On day 6, participants also filled out the Vaccine HCRU survey (Figure 1). 

### 2.2. Participants

Participants voluntarily seeking the COVID-19 vaccine were recruited from primary care or specialty care clinics. Eligible participants were aged ≥18 to ≤65 years, receiving a first, second, or booster dose of a COVID-19 vaccine (US: BNT162b2, mRNA-1273, Ad26.COV2.S, NVX-CoV2373; Canada: BNT162b2, mRNA-1273, Ad26.COV2.S, NVX-CoV2373, AZD1222, CoVLP), were employed (working for pay) for ≥20 h per week, were scheduled to work ≥3 days in the 6 days post-dose, and had access to a smartphone, tablet, or computer (to complete the daily study diary). 

Exclusion criteria included participation in investigational product research in the 45 days prior to study enrollment, confirmed/suspected immunocompromising condition or chronic administration of immunosuppressant medications in the 3 months prior to study enrollment, history of severe allergic reaction to prior COVID-19 vaccines, receipt of any other vaccine 1 week prior to study enrollment, and plans to receive a co-administered vaccine on day 0 or any other vaccine in the 6 days post–study vaccination. All participants provided written informed consent prior to study vaccine receipt and survey completion.

### 2.3. Survey Instruments

A screener/vaccine information survey (inclusion/exclusion criteria, prior/current COVID-19 vaccines) and baseline questionnaire (standard baseline demographics/medical history questions, employment status) were given on day 0. On days 1–6, a daily diary was completed by the participant to assess whether they were scheduled to work, the number of hours worked, the number of missed work hours, productivity ratings (at work and in regular activities), and vaccine-related reactogenicity symptoms. Day 0 absenteeism and presenteeism (post-vaccination) were assessed on day 1. The Absenteeism and Presenteeism Diary portion of this assessment was adapted from the Work Productivity and Activity Impairment Questionnaire: Specific Health Problem version 2.0. To adapt the questionnaire, the first question asking about employment status was removed, and a recall period of 24 h was used. The Vaccine Symptoms Diary measured 11 patient-reported symptoms to assess reactogenicity, with a 24 h recall period (local and systemic events). On day 6, a Vaccine HCRU survey was administered to assess medication use, unplanned doctor visits, urgent healthcare center visits, hospital emergency department visits, and hospitalizations related to COVID-19 vaccine events (7-day recall period).

### 2.4. Objectives

The goal of this study was to compare the burden of common COVID-19 vaccine side effects (i.e., reactogenicity) on vaccine-related work absenteeism and presenteeism and loss of daily (non-work) activity for NVX-CoV2373 versus other authorized/approved vaccines in a real-world setting. The primary endpoint was the absolute difference in the percentage of participants with an overall work impairment score of ≥50% for any day during the 6-day post-vaccination period. Overall work impairment was defined as the summation of a participant′s absenteeism rate (hours missed/total hours scheduled) and presenteeism rate (hours worked/total hours scheduled multiplied by percent impairment) for each day reported working for NVX-CoV2373 versus mRNA vaccines after the booster dose (regardless of prior dose vaccine and prior COVID-19 disease status). A key secondary objective/endpoint was to assess the percentage of participants with an overall work impairment score of ≥50% for any day during the 6-day post-vaccination period after a primary series dose.

Other key endpoints were the mean percentage of overall work impairment, mean percentage of work-time missed, mean percentage of impairment while working (as rated by participants on a scale of 0 to 10 daily, averaged across the post-vaccination period and then divided by 10), mean percentage of activity impairment (as rated by participants on a scale of 0 to 10), mean percentage of hours with diminished/impaired performance at work (i.e., worked any amount of time with impaired performance), and difference in healthcare utilization (i.e., over-the-counter and prescription medication use; office, urgent care, and emergency department visits; and hospital/intensive care unit nights).

Exploratory/additional endpoints for NVX-CoV2373 versus mRNA booster doses or primary series in the 6 days post-vaccination (regardless of prior dose vaccine) included reactogenicity, rate difference in full-day impairment while working (full calendar workday with diminished performance), impairment while working rate (defined as days with any amount of time at work with diminished performance), absolute number of hours at work with diminished performance after the booster dose, and difference in the percentage of hours worked over the 6-day post-vaccination period with any diminished performance (out of the total number of hours worked). Impairment of non-work activities was also examined. Endpoints were also examined for differences between NVX-CoV2373 and mRNA vaccines by the brand after a booster dose or primary series.

### 2.5. Statistics

#### Sample Size and Analyses

Two main subpopulations were powered for and defined in this study: the Booster Dose Population, which consisted of study participants who received any booster dose of NVX-CoV2373 or an mRNA vaccine (i.e., BNT162b2 or mRNA-1273), regardless of which vaccine was used for the prior doses and prior COVID-19 disease status, and the Primary Series Population, which consisted of study participants who received their first or second dose of NVX-CoV2373 or an mRNA vaccine, regardless of which vaccine was used for the first dose and prior COVID-19 disease status. The sample size was determined based on the ability to power the primary objective In the booster dose population, as well as the ability to have an adequate sample for the key secondary objective in the primary series population and exploratory objectives. Assuming a 1:3 NVX-CoV2373:mRNA recruitment ratio, a Booster Dose Population of ≥289 NVX-CoV2373 participants and 866 mRNA vaccine participants was targeted for enrollment to detect an absolute difference of 10% between NVX-CoV2373 (20%) and an mRNA vaccine (30%) when the primary objective was evaluated at 80% power using a two-sided Fisher’s exact test with a 0.05 significance level, including a 22% adjustment for dropout. Assuming a 1:1 NVX-CoV2373:mRNA recruitment ratio, a Primary Series Population of ≥346 participants (173 per group) was targeted for enrollment to detect a difference of 15% in the percentage of participants with work impairment between NVX-CoV2373 (20%) and an mRNA vaccine (35%) when the key secondary objective was evaluated at 80% power using a Fisher’s exact test with a 0.05 significance level, including a 15% adjustment for dropout. Because vaccine doses were treated independently, 1501 individuals were required to power the primary (Booster Dose Population) and key secondary (Primary Series Population) objectives. A total sample size of 1750 participants was deemed feasible based on practical considerations, with 249 individuals included for over-sampling to account for any increases in the dropout rate and other vaccines.

The analyses consisted of (1) descriptive analyses on the entire population and (2) comparative analyses on an analysis sample defined as vaccine recipients who were classified as having worked ≥3 days during the 6-day post-vaccination period per the Absenteeism and Presenteeism Diary. Descriptive analyses were conducted on responses to the baseline survey and daily diary. Comparative analyses of primary and secondary endpoints were adjusted for confounding using propensity scores [29]. Propensity scores were assigned to each participant based on a select group of demographic/clinical characteristics identified using standardized differences (Supplemental Methods) [30,31,32]. The comparative analyses were weighted using stabilized inverse probability of treatment weights [33], developed independently for the 6-day post-vaccination period and days 1 and 2 post-vaccination periods, for the Booster Dose and Primary Series Populations (example shown in Figures S2 and S3 for booster at day 6). To account for areas of non-overlap in the propensity score distributions, sensitivity analyses utilizing asymmetric trimming of the propensity scores were conducted (participants were removed from the analysis if their propensity score was at or below the 1st percentile associated with NVX-CoV2373 participants or at or above the 9th percentile associated with mRNA vaccine participants). 

Specific descriptive and comparative results were further analyzed by the following: specific diary day (i.e., day 0 to day 6), diary days 1 and 2 only, prior COVID-19 status (i.e., with or without prior COVID-19), mRNA vaccine brand (i.e., BNT162b2 or mRNA-1273), and country (i.e., the US or Canada). The results were not adjusted for multiple comparisons.

## 3. Results

### 3.1. Participants

**Figure 2:**
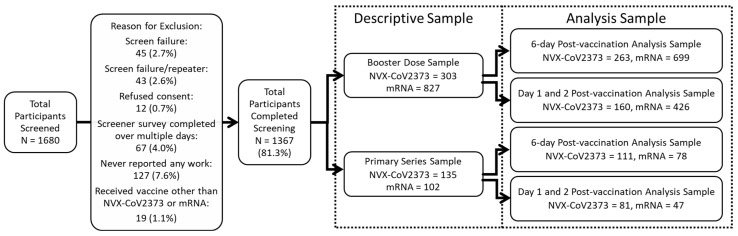
Participant screening and inclusion. The descriptive sample includes all eligible participants. Comparative analyses for the 6-day post-vaccination analysis sample comprised participants that worked at least 3 of the 6 days post-vaccination; the Day 1 and 2 analysis samples comprised participants that worked on both days post-vaccination. Participants can be in both the 6-day and Day 1 and 2 post-vaccination analysis samples.

This study was conducted between July 2022 and March 2023. A total of 1680 participants were screened; of these, 1367 were eligible (88 were screen failures/repeaters, and 12 refused consent). A total of 1130 participants received a booster dose during the study period (237 received a primary series). Of the 1130 participants with a booster dose, 303 received NVX-CoV2373, and 827 received an mRNA booster (Figure 2). 

For the Booster Dose Population (n = 1130), baseline demographics were well-balanced between vaccine groups, with a few exceptions. The mean (standard deviation) age was 38.9 (11.8) years for recipients of NVX-CoV2373 and 40.1 (13.0) years for recipients of an mRNA vaccine. The race and ethnicity of participants differed between booster types: 13.2% who received an NVX-CoV2373 booster were Asian compared with 22.9% who received an mRNA booster, and 50.8% who received an NVX-CoV2373 booster were identified as Hispanic compared with 25.0% who received an mRNA booster. Recipients of mRNA boosters more frequently had a college education or above (NVX-CoV2373: 42.6%; mRNA vaccine: 61.3%), were executives/professionals (22.4% vs. 38.0%), and had an annual income ≥ $50,000 (30.7% vs. 42.0%); however, there was a sizeable percentage of participants who chose not to answer these questions (Table 1). 

For clinical characteristics among booster recipients, 39.3% who received an NVX-CoV2373 booster and 52.4% who received an mRNA booster had been previously diagnosed with COVID-19. The percentage of participants reporting a medical condition that put them at high risk for severe COVID-19 was relatively low (NVX-CoV2373: 6.3%; mRNA vaccine: 5.4%) (Table 1).

Of the 237 participants in the Primary Series Population, 135 received NVX-CoV2373 and 102 received an mRNA vaccine. Baseline demographics and clinical characteristics were generally similar between vaccine groups, although some exceptions were noted. Specifically, a smaller proportion of NVX-CoV2373 versus mRNA vaccine recipients were White (17.0% vs. 31.4%); differences were also noted for certain annual income brackets (Table 1).

### 3.2. Responses to Vaccine-Related Local and Systemic Reactogenicity Symptoms

**Figure 3:**
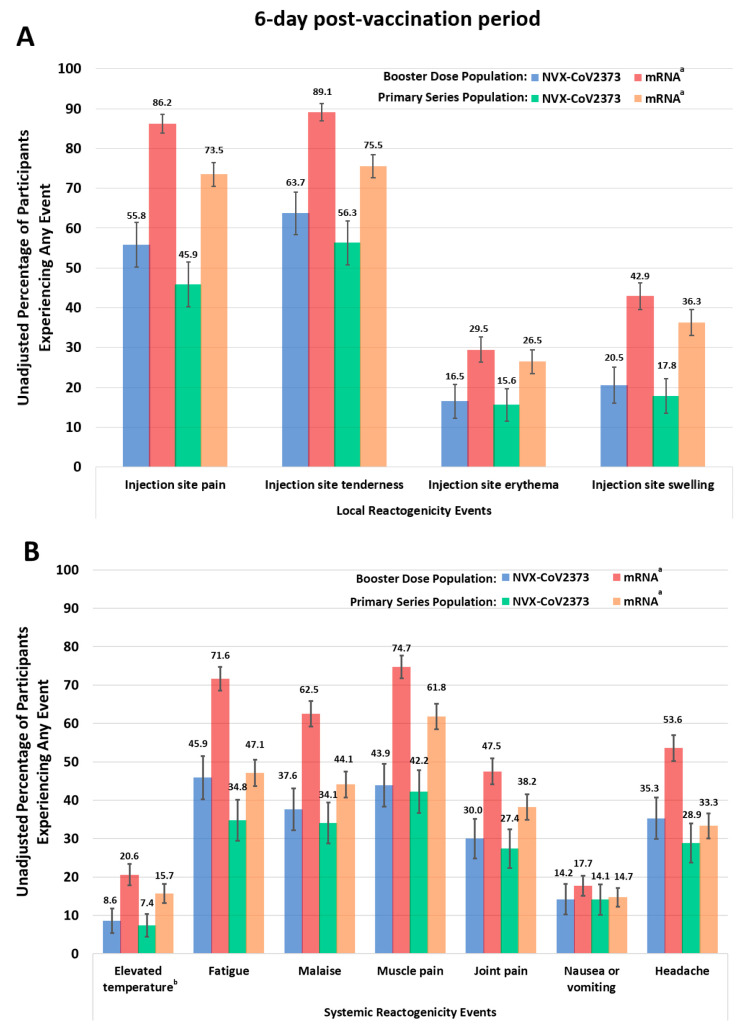
Responses to the Vaccine Symptoms Diary ((**A**), local, and (**B**), systemic reactogenicity) in the 6-day post-vaccination period for either the booster or primary series doses. Participants experienced an event if a grade 1 or worse reactogenicity event was reported for any day during the 6-day post-vaccination period. ^a^ Individuals received either BNT162b2 or mRNA-1273. ^b^ Temperatures at or above 100.4 °F were considered elevated.

Overall, based on the descriptive sample, lower unadjusted rates of local and systemic reactogenicity symptoms were reported for NVX-CoV2373 than for mRNA vaccine recipients (booster or primary series) (Figure 3, Table S1). Additionally, a larger proportion of reported events were grade 1 (mild) following receipt of NVX-CoV2373 than an mRNA vaccine.

For the Booster Dose descriptive sample, a smaller unadjusted proportion of NVX-CoV2373 booster recipients reported injection site pain that required the use of nonprescription pain relievers or interfered with activity (9.2% across the 6-day post-vaccination period; 7.8% over days 1 and 2) than did mRNA recipients (29.1% vs. 28.9%). A similar trend was seen over days 1–2 for injection site tenderness, elevated temperature, fatigue, malaise/feeling unwell, muscle pain, and joint pain (Figure S4).

For the Primary Series descriptive sample, a smaller unadjusted proportion of NVX-CoV2373 recipients reported injection site pain (45.9% across the 6-day post-vaccination period; 45.7% over days 1 and 2) than mRNA vaccine recipients (73.5% vs. 72.4%) (Figure 3, Table S1). A smaller unadjusted proportion of NVX-CoV2373 vaccine recipients reported mild swelling at the injection site (14.1% across the 6-day post-vaccination period; 12.4% over days 1 and 2) than mRNA recipients (27.5% vs. 28.6%). Similar trends were noted over days 1–2 for injection site tenderness, elevated temperature, fatigue, malaise/feeling unwell, muscle pain, and joint pain (Figure S4, Table S1).

### 3.3. Overall Work Impairment after Vaccination

#### 3.3.1. Participants with an Overall Work Impairment Score of ≥50%

**Figure 4:**
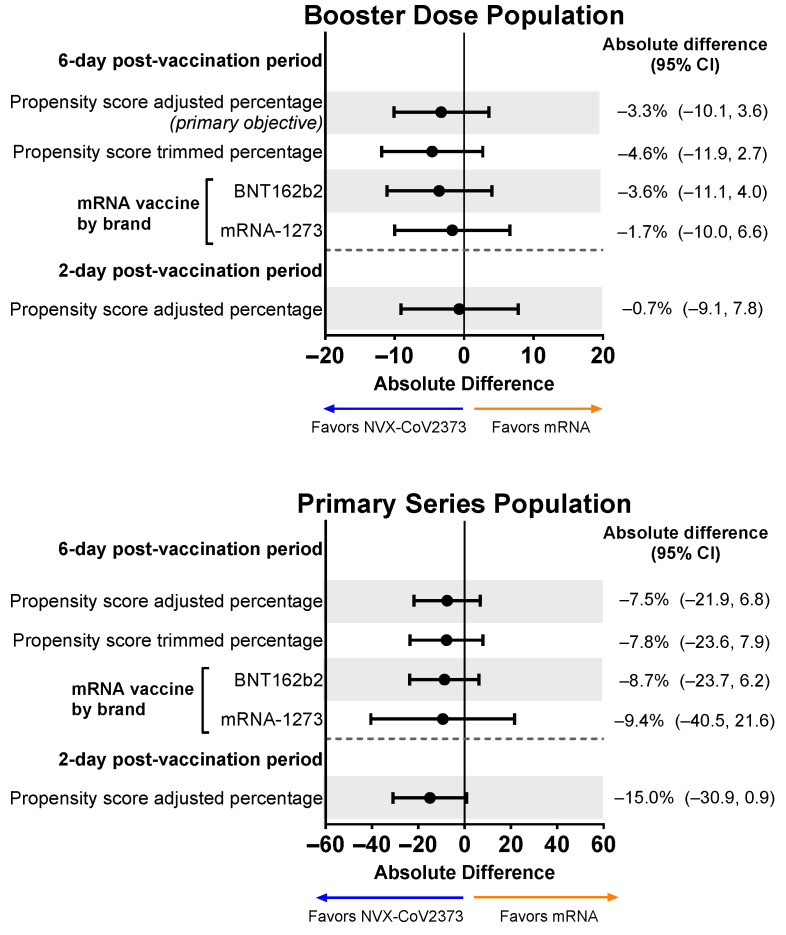
Forest plots showing estimated differences (NVX-CoV2373—mRNA) in the proportion of participants reporting overall work impairment scores of ≥50% for ≥1 day during the 6-day post-vaccination period. Results are shown for the Booster Dose and Primary Series Populations. Percentages and 95% CIs derive from a propensity score-weighted logistic regression for the comparison of the NVX-CoV2373 and mRNA vaccine groups. Note: The scales for the absolute difference differ for the Booster Dose and Primary Series Populations.

The proportion of individuals in the Booster Dose Population reporting an overall work impairment score of ≥50% for ≥1 day during the 6-day post-vaccination period in the descriptive analysis was 38.8% for recipients of NVX-CoV2373 and 41.6% for recipients of an mRNA vaccine. For the primary objective, when restricting to the 6-day post-vaccination analysis sample and adjusting for propensity scores, the magnitude of the difference increased in favor of NVX-CoV2373 (absolute difference: −3.3%; 95% CI, −10.1% to 3.6%). After asymmetric trimming was performed, 43 NVX-CoV2373 recipients and 21 mRNA vaccine recipients were removed from the analysis. This sensitivity analysis further increased the magnitude of the difference in favor of NVX-CoV2373 (NVX-CoV2373: 37.5%; mRNA vaccine: 42.0%; absolute difference: −4.6%; 95% CI, −11.9% to 2.7%) (Figure 4). Similar results were seen when comparing NVX-CoV2373 to each individual mRNA vaccine (i.e., BNT162b2 or mRNA-1273), although it should be noted that the majority (60.7%) of mRNA booster recipients received BNT162b2. For participants working in the 2 days post-vaccination (NVX-CoV2373: n = 160; mRNA vaccine: n = 426), the direction of the difference also showed NVX-CoV2373 recipients to have less work impairment; however, after adjusting for propensity scores, the magnitude of the difference was not as pronounced as that seen across the 6-day post-vaccination period.

For the Primary Series Population, the proportion of participants with an overall work impairment score of ≥50% for ≥1 day during the 6-day post-vaccination period in the descriptive analysis was 36.0% for recipients of NVX-CoV2373 and 44.9% for recipients of an mRNA vaccine. Propensity score-adjusted and trimmed percentages demonstrated a slight favoring of NVX-CoV2373, although 95% CIs for all differences crossed 0 (Figure 4).

#### 3.3.2. Mean Percentages of Overall Work Impairment

**Figure 5:**
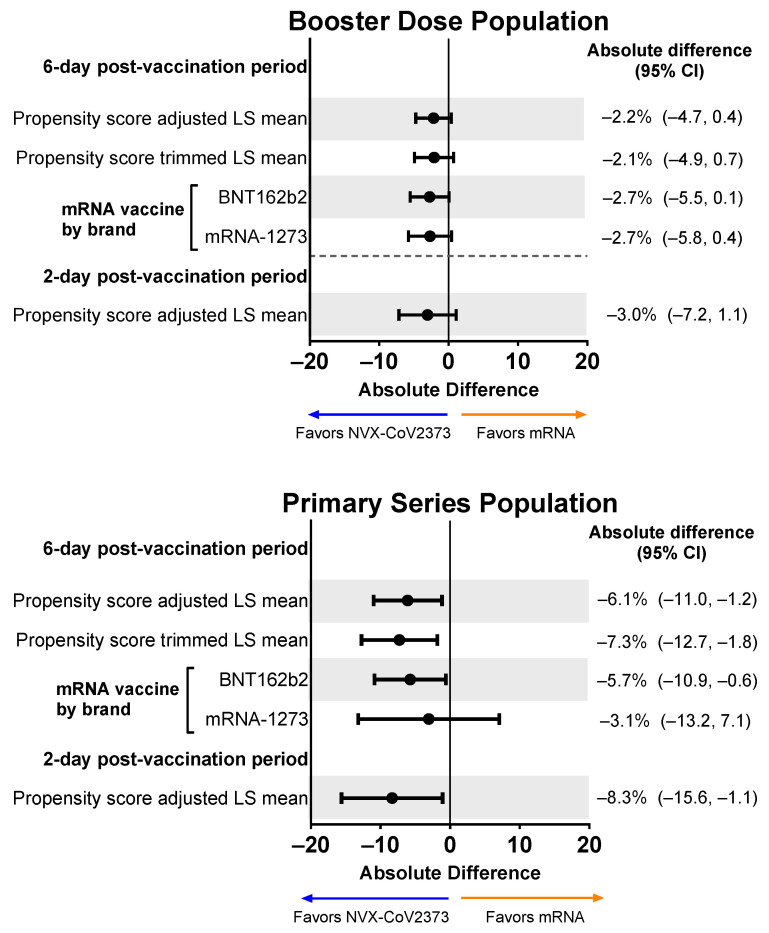
Forest plots showing estimated differences (NVX-CoV2373—mRNA) of the mean percentage of overall work impairment. Results are shown for the Booster Dose and Primary Series Populations. Overall work impairment was a summary score estimated using the sum of hours reported (hours missed and hours worked) and the average of the impairment scores from the daily diary collected over the post-vaccination period. LS means and 95% Cis were derived from a propensity score weighted ANOVA model for the comparison of the NVX-CoV2373 and mRNA vaccine groups. LS, least squares.

For the Booster Dose Population, the mean percentage of overall work impairment over the 6-day post-vaccination period was 15.9% for recipients of NVX-CoV2373 and 18.6% for recipients of an mRNA vaccine. After adjusting for propensity scores, the overall work impairment experienced by recipients in both vaccine groups was comparable (absolute difference: −2.2%; 95% CI, −4.7% to 0.4%) (Figure 5). This trend was consistent over days 1 and 2 of the study and for both brands of mRNA vaccines (i.e., BNT162b2 or mRNA-1273). Similar results were seen in the Primary Series Population. 

### 3.4. Missed Work after Vaccination

**Figure 6:**
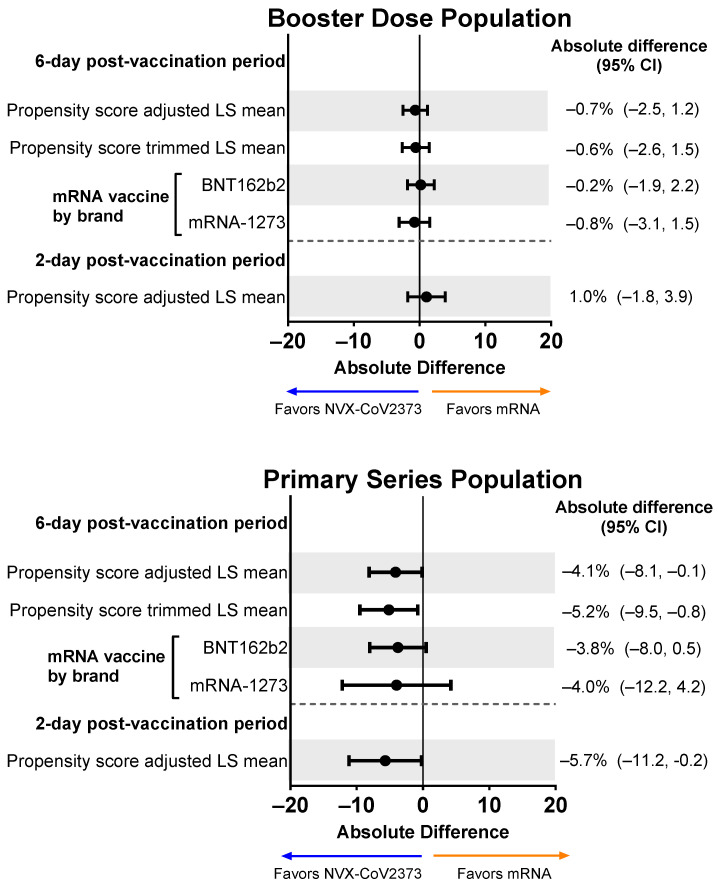
Forest plots showing estimated differences (NVX-CoV2373—mRNA) of the mean percentage of work time missed. Results are shown for the Booster Dose and Primary Series Populations. Percent of work missed was a summary score estimated using the sum of hours reported (hours missed and hours worked) from the daily diary collected over the post-vaccination period. LS means and 95% CIs derive from a propensity score weighted ANOVA regression for the comparison of the NVX-CoV2373 and mRNA vaccine groups. LS, least squares.

Participants in both vaccine booster groups reported missing an average of 10% of their work time after vaccination (NVX-CoV2373, 9.4%; mRNA vaccine, 9.6%). Following adjustment, the mean percentage difference was minimal (−0.7%; 95% CI, −2.5% to 1.2%), and the mean percentage of work time missed was similar when NVX-CoV2373 was compared to each individual mRNA vaccine (i.e., BNT162b2 or mRNA-1273) (Figure 6). Results for the Primary Series Population showed a greater absolute difference in LS Mean, with the propensity score–adjusted LS Mean difference being statistically significant.

### 3.5. Impairment While Working after Vaccination

**Figure 7:**
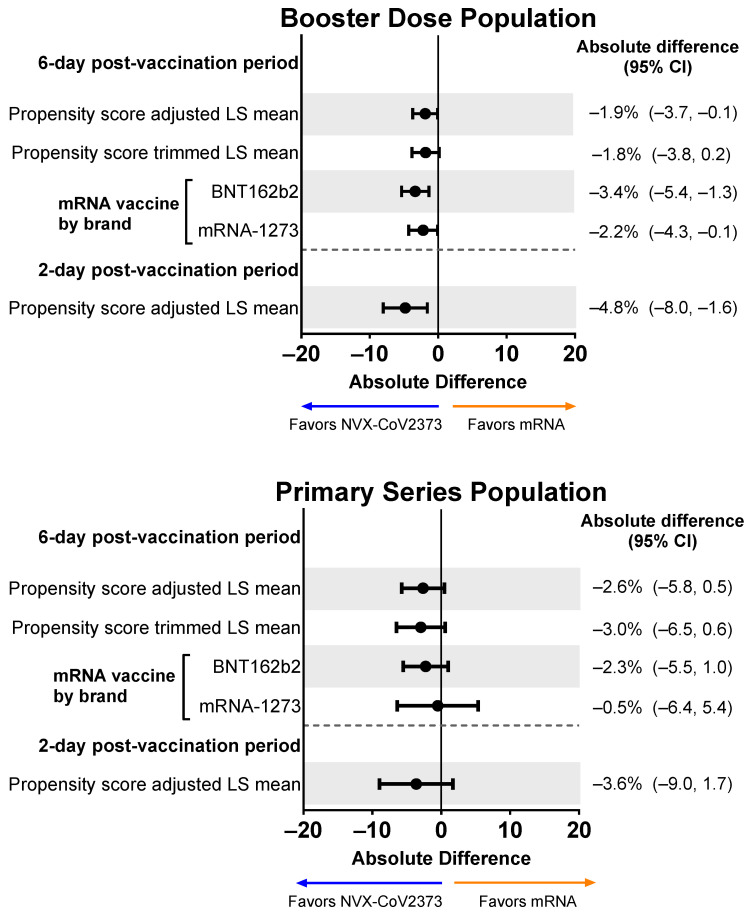
Forest plots showing estimated differences (NVX-CoV2373—mRNA) in the mean percentage of impairment while working. Results are shown for the Booster Dose and Primary Series Populations. Impairment while working was estimated by averaging the impairment scores (rated 0–10) collected from the daily diary over the post-vaccination period. LS means and 95% CIs derive from a propensity score weighted ANOVA regression for the comparison of the NVX-CoV2373 and mRNA vaccine groups. LS, least squares.

Those who received an NVX-CoV2373 booster experienced less impairment while working than participants who received an mRNA booster (Figure 7). When adjusted for confounding variables, the difference favoring NVX-CoV2373 persisted (mean absolute difference across the 6-day post-vaccination period: −1.9%, 95% CI, −3.7% to −0.1%; mean absolute difference over days 1 and 2: −4.8%, 95% CI, −8.0% to −1.6%). Results were similar for the Primary Series Population.

### 3.6. Non-Work Activity Impairment after Vaccination

**Figure 8:**
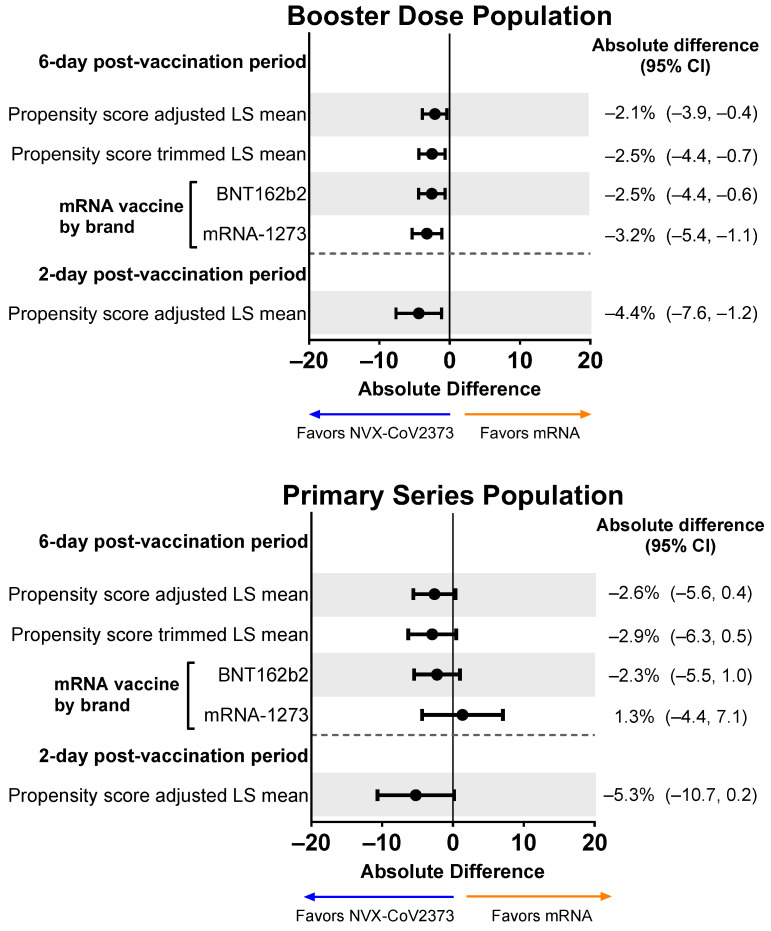
Forest plots showing estimated differences (NVX-CoV2373—mRNA) of the mean non-work activity impairment. Results are shown for the Booster Dose and Primary Series Populations. Impairment in daily activities was estimated by averaging the impairment scores (rated 0–10) collected from the daily diary over the post-vaccination period. LS means and 95% CIs derive from a weighted propensity score ANOVA regression for the comparison of the NVX-CoV2373 and mRNA vaccine groups. LS, least squares.

Based on the symptom diary, NVX-CoV2373 booster recipients experienced less activity impairment compared with mRNA booster recipients, as indicated by lower activity impairment scores (including when adjusted) across the 6-day post-vaccination period and over days 1 and 2 (Figure 8). NVX-CoV2373 booster recipients had lower mean activity impairment than recipients of either of the mRNA boosters (i.e., BNT162b2 or mRNA-1273). For the Primary Series Population, NVX-CoV2373 recipients trended toward less activity impairment than mRNA vaccine recipients. 

### 3.7. Diminished Work Performance after Vaccination

**Figure 9:**
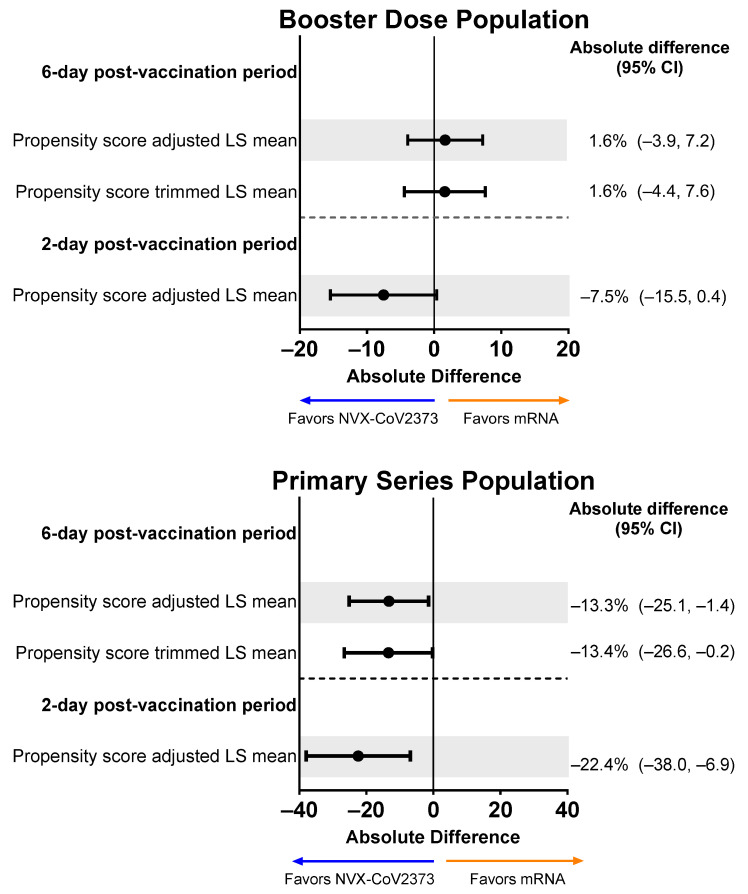
Forest plots showing estimated differences (NVX-CoV2373—mRNA) in the mean percentage of work hours with diminished performance. Results are shown for the Booster Dose and Primary Series Populations. Diminished work performance was defined as the number of hours worked with impairment out of the total number of hours worked over the post-vaccination period. LS means and 95% CIs derive from a propensity score weighted ANOVA regression for the comparison of the NVX-CoV2373 and mRNA vaccine groups. Note: The scales for the absolute difference differ for the Booster Dose and Primary Series Populations. LS, least squares.

In the Booster Dose Population, the mean percentage of work hours with diminished performance due to any level of impairment was lower for recipients of NVX-CoV2373 than an mRNA vaccine over days 1 and 2 following propensity score adjustment (Figure 9). Following propensity score adjustment of the Primary Series Population, NVX-CoV2373 versus mRNA vaccine recipients displayed a lower mean percentage of hours with diminished work performance due to any level of impairment across the 6-day post-vaccination period and over days 1 and 2.

### 3.8. Healthcare Utilization

Use of over-the-counter and prescription medications was low and similar for recipients of NVX-CoV2373 and an mRNA vaccine, and few study participants sought medical care post-vaccination (Table S2). Due to the low reported use of medications and medical care, no comparative modeling was performed.

## 4. Discussion

This study adds to the growing body of research measuring the frequency of COVID-19 vaccine-related side effects and their associated burden. In the 2019nCoV-406 study, a greater percentage of participants who received an mRNA booster reported greater local and systemic reactogenicity events, including injection-site pain, tenderness, elevated temperature, fatigue, malaise/feeling unwell, muscle pain, and joint pain, as compared to NVX-CoV2373. As shown in Figure 3, the percentage of individuals who experienced vaccine-related side effects was similar irrespective of whether they were administered as a booster or primary series. The use of medications and medical care for post-vaccination symptoms was low for all groups. 

The rates of post-vaccination symptoms reported in this study are similar to those reported in the V-safe study of mRNA vaccines [34,35]. In addition, the reactogenicity of the NVX-CoV2373 booster observed in 2019nCoV-406 is consistent with the percentage reported in the US FDA Healthcare Provider Fact Sheet [36]. A recent meta-analysis of reactogenicity among COVID-19 vaccine types found mRNA vaccines to be more reactogenic and to lead to greater impairment than inactivated vaccines [37,38]. The observed pattern of greater reactogenicity following mRNA compared to NVX-CoV2373 from 2019nCoV-406 is in agreement with previous investigations of COVID-19 vaccine safety, including the Oxford COV-BOOST trial and a National Institute for Allergy and Infectious Diseases (NIAID) of the National Institutes of Health (NIH) funded booster study [39,40,41,42]. Notably, the referenced clinical studies were small and not designed to compare vaccine-associated side effects by vaccine type. Despite this, the consistency of reported reactogenicity event trends across various booster and primary series studies is clear [37,39,40,41,43,44]. Here, 2019nCoV-406 offers new real-world insights and an examination of human behavior associated with differences in reactogenicity. Notably, some are calling for healthcare workers and certain other professionals to receive vaccines of lower reactogenicity [35]. Based on the findings of this and prior studies [37,39,40,41,42,43,44], NVX-CoV2373 may provide a less reactogenic option for these individuals.

The goal of this prospective, real-world observational study was to compare the impact of NVX-CoV2373 and mRNA vaccine-related reactogenicity on work absenteeism, presenteeism, and activity impairment. For the primary and key secondary endpoints of the percentage of participants with an overall work impairment score of ≥50% for ≥1 day during the 6-day post-vaccination period, the results favored NVX-CoV2373 over mRNA vaccines when used as a booster or for the primary series. However, there were few significant differences between vaccine types, as the 95% CIs for the differences often contained 0. In the Primary Series Population, a difference was seen between recipients of NVX-CoV2373 and an mRNA vaccine in the percentage of missed work. For the primary series, NVX-CoV2373 recipients reported lower overall work impairment, but the difference was only significant when NVX-CoV2373 was compared with BNT162b2 (absolute difference [95% CI]: −5.7% [−10.9%, −0.6%]). This may be a possible consequence of the small number of participants who received mRNA-1273 for their primary series (n = 11), limiting the ability to detect differences by brand. A similar trend was seen in booster recipients, but the difference was smaller and not significant (absolute difference [95% CI]: −2.7% [−5.5%, 0.1%]). Although mRNA booster recipients experienced more severe reactogenic events and greater activity impairment than NVX-CoV2373 booster recipients, they still attended work, thus affecting the ability to detect statistically significant differences on the primary endpoint. This difference in response to COVID-19 vaccine reactogenicity could be due to habituation; that is, as individuals receive additional COVID-19 vaccines, they become more tolerant of vaccine-related side effects and are therefore less likely to miss workdays.

There were, however, some endpoints in which significant differences were detected between vaccine types. For the Booster Dose Population, the percentage of impairment while working at days 1–2 post-vaccination and the percentage of activity impairment were significantly favoring NVX-CoV2373 versus mRNA booster recipients. For the Primary Series Population, NVX-CoV2373 was significantly favored over mRNA for the percentage of overall work impairment, percentage of work time missed at days 1–2 post-vaccination, and percentage of work hours with diminished performance due to any level of impairment. An association between types of COVID-19 vaccines and impairment of work and other activities has been previously demonstrated. In a study comparing ChAdOx with mRNA vaccines, an inability to work was reported most frequently after a first dose of ChAdOx (33.6% over days 1–2; 10% over ≥3 days) and a second dose of mRNA-1273 (29.6% over days 1–2; 9.2% over ≥3 days) [35]. Although COVID-19 and long COVID have a greater impact on local and systemic events and work productivity than vaccines [45,46], a vaccine of limited reactogenicity is critical for reducing the impact on work and daily activities. 

### Limitations

Study participants were derived from a convenience sample and thus may not be representative of all populations receiving or healthcare practices administering COVID-19 vaccines. Due to low enrollment at some sites, there was a concentration of participants with particular demographic characteristics (e.g., race/ethnicity) who received a particular vaccine (e.g., BNT162b2). Due to the timing of vaccine availability, the majority of mRNA vaccine recipients were enrolled in mid-to-late 2022, whereas the majority of NVX-CoV2373 recipients were enrolled in early 2023. The results of this study are subject to potential selection bias and responder bias; whether individuals who were ineligible to participate (e.g., those lacking internet access) would have reported different outcomes is unknown. With the noted imbalance of some variables between vaccine platforms, comparing unadjusted data should be undertaken with caution. However, these concerns are inherent to all non-interventional, real-world studies. As with all studies that require participants to self-report outcomes and behaviors, completeness, and accuracy can be a concern. Although study participants were asked to complete the daily questions at the same time of day, they could have accessed and completed the daily questions during an 8 h window. Some participants may have misinterpreted questions about hours worked and hours missed per day, resulting in overreporting of hours intended to be worked. 

## 5. Conclusions

In conclusion, participants who received an NVX-CoV2373 versus mRNA booster exhibited overall lower reactogenicity. Although this study’s primary endpoint (percentage of participants with an overall work impairment score of ≥50% on ≥1 day in the 6-day post-vaccination period) did not show significant differences, the results suggest that NVX-CoV2373 booster recipients trended toward having less overall work impairment than mRNA booster recipients. While data from this real-world study suggest that NVX-CoV2373 may be associated with more favorable outcomes relative to mRNA vaccines, additional research is needed to confirm these findings.

## Figures and Tables

**Table 1:** Participant demographics and clinical characteristics in the Booster Dose Population.

	Booster Dose Population		Primary Series Population	
Parameter	NVX-CoV2373 (n = 303)	mRNA Vaccine ^a^ (n = 827)	NVX-CoV2373 (n = 135)	mRNA Vaccine ^a^ (n = 102)
**Age, mean (SD) years**	38.9 (11.8)	40.1 (13.0)	37.8 (11.0)	34.9 (12.0)
**Gender identity, n (%)**				
Female	156 (51.5)	469 (56.7)	65 (48.1)	62 (60.8)
Male	142 (46.9)	355 (42.9)	69 (51.1)	40 (39.2)
Genderfluid	1 (0.3)	0	0	0
Nonbinary	2 (0.7)	3 (0.4)	0	0
A gender identity not listed	0	0	0	0
Prefer not to answer	2 (0.7)	0	1 (0.7)	0
**Race/ethnicity ^b^, n (%)**				
African American or Black	33 (10.9)	77 (9.3)	16 (11.9)	17 (16.7)
Alaska Native, American Indian, or Native American	2 (0.7)	4 (0.5)	2 (1.5)	0
Asian ^c^	40 (13.2)	189 (22.9)	3 (2.2)	0
Hispanic, Latin American, or Latinx	154 (50.8)	207 (25.0)	98 (72.6)	67 (65.7)
Middle Eastern or North African ^d^	5 (1.7)	21 (2.5)	0	0
Native Hawaiian or Pacific Islander ^e^	6 (2.0)	75 (9.1)	0	1 (1.0)
White	152 (50.2)	278 (33.6)	23 (17.0)	32 (31.4)
Race or ethnicity not listed	3 (1.0)	13 (1.6)	0	0
Prefer not to answer	6 (2.0)	5 (0.6)	2 (1.5)	1 (1.0)
**Education level, n (%)**				
Less than secondary (high) school graduation	4 (1.3)	15 (1.8)	3 (2.2)	3 (2.9)
Secondary (high) school diploma or equivalent	70 (23.1)	145 (17.5)	73 (54.1)	41 (40.2)
Some college/post-secondary school	66 (21.8)	153 (18.5)	24 (17.8)	32 (31.4)
College degree/post-secondary certificate, diploma, or degree	80 (26.4)	305 (36.9)	21 (15.6)	19 (18.6)
Professional, advanced, or graduate degree	49 (16.2)	202 (24.4)	12 (8.9)	5 (4.9)
Prefer not to answer	34 (11.2)	7 (0.8)	2 (1.5)	2 (2.0)
**Work from home or work outside of the home, n (%)**				
Work from home	55 (18.2)	196 (23.7)	18 (13.3)	7 (6.9)
Work outside the home	237 (78.2)	616 (74.5)	115 (85.2)	93 (91.2)
Prefer not to answer	11 (3.6)	15 (1.8)	2 (1.5)	2 (2.0)
**Type of job, n (%)**				
Executive, administrator, or senior manager	10 (3.3)	57 (6.9)	3 (2.2)	1 (1.0)
Professional	58 (19.1)	257 (31.1)	11 (8.1)	8 (7.8)
Technical support	30 (9.9)	63 (7.6)	7 (5.2)	5 (4.9)
Sales	37 (12.2)	59 (7.1)	17 (12.6)	16 (15.7)
Clerical and administrative support	18 (5.9)	91 (11.0)	6 (4.4)	8 (7.8)
Service occupation	44 (14.5)	118 (14.3)	46 (34.1)	38 (37.3)
Precision production and crafts worker	8 (2.6)	17 (2.1)	23 (17.0)	4 (3.9)
Operator or laborer	23 (7.6)	84 (10.2)	17 (12.6)	15 (14.7)
Active military	1 (0.3)	0	0	0
Prefer not to answer	74 (24.4)	81 (9.8)	5 (3.7)	7 (6.9)
**Annual income from all employment, n (%)**				
$1 to $9999	5 (1.7)	25 (3.0)	7 (5.2)	6 (5.9)
$10,000 to $24,999	37 (12.2)	114 (13.8)	26 (19.3)	22 (21.6)
$25,000 to $49,999	88 (29.0)	216 (26.1)	36 (26.7)	42 (41.2)
$50,000 to $99,999	63 (20.8)	223 (27.0)	20 (14.8)	11 (10.8)
$100,000 to $199,999	29 (9.6)	106 (12.8)	3 (2.2)	0
$200,000 to $499,999	1 (0.3)	17 (2.1)	0	0
$500,000 or more	0	1 (0.1)	0	0
Prefer not to answer	80 (26.4)	125 (15.1)	43 (31.9)	21 (20.6)
**Essential worker, n (%)**				
Yes	134 (44.2)	390 (47.2)	73 (54.1)	56 (54.9)
No	137 (45.2)	400 (48.4)	36 (26.7)	33 (32.4)
Prefer not to answer	32 (10.6)	37 (4.5)	26 (19.3)	13 (12.7)
**Prior COVID-19 diagnosis, n (%)**	**119 (39.3)**	**433 (52.4)**	47 (34.8)	49 (48.0)
**Medical condition that puts participant at high risk for severe COVID-19 ^b^, n (%)**	**19 (6.3)**	**45 (5.4)**	1 (0.7)	4 (3.9)
Diabetes	6 (31.6)	21 (46.7)	0	0
Hypertension	7 (36.8)	13 (28.9)	0	0
Heart disease	2 (10.5)	8 (17.8)	0	0
Respiratory conditions	5 (26.3)	11 (24.4)	0	1 (25.0)
Other	5 (26.3)	13 (28.9)	1 (100)	3 (75.0)
**Booster dose, n (%)**				
First	184 (60.7)	309 (37.4)	–	–
Second or later	119 (39.3)	518 (62.6)	–	–
**mRNA vaccine type, n (%)**				
Monovalent	–	652 (78.8)	-	102 (100)
Bivalent	–	175 (21.2)	-	0

SD = standard deviation. ^a^ Individuals received either BNT162b2 or mRNA-1273. ^b^ The categories for these variables were not mutually exclusive (participants could have listed more than one). ^c^ Includes participants who identified as Chinese, South Asian (e.g., East Indian, Pakistani, or Sri Lankan), Southeast Asian (e.g., Vietnamese, Cambodian, Laotian, or Thai), Korean, or Japanese. ^d^ Includes participants who identified as Middle Eastern, North African, Arab, or West Asian (e.g., Iranian, or Afghan). ^e^ Includes participants who identified as Native Hawaiian, Pacific Islander, or Filipino.

## Data Availability

Data are available on request due to privacy restrictions. The data presented in this study are available in aggregate form on request to the corresponding author. The data are not publicly available due to privacy requirements.

## Supplementary Materials

The following supporting information can be downloaded at: https://www.mdpi.com/article/10.3390/vaccines12010083/s1, Figure S1: Baseline survey; Figure S2: Standardized mean differences plot for the 6-day post-vaccination booster sample; Figure S3: Propensity score distribution for the 6-day post-vaccination booster sample; Figure S4: Responses to Vaccine Symptoms diary (reactogenicity) for the 2-day post-vaccination period for the Booster Dose and Primary Series Populations; Table S1: Percentage of any reactogenicity events in the 6-day or 2-day post-vaccination period for either the booster or primary series doses; Table S2: Healthcare resource utilization among booster dose recipients over the 6-day post-vaccination period.
