# Supplementary material for: Burden and Impact of Reactogenicity among Adults Receiving COVID-19 Vaccines in the United States and Canada: Results from a Prospective Observational Study
Source: Vaccines (Basel). 2024 Jan 13;12(1):83. doi: 10.3390/vaccines12010083 (PMC10821469; doi:10.3390/vaccines12010083)
Supplement: Supplementary file 1 [file vaccines-12-00083-s001.zip › vaccines-2784621-supplementary.pdf]

vaccines

SUPPLEMENT

Table of Contents **Page**

Supplemental Methods……………………………………………………………………………………………………….2
Table S1. Percentage of any reactogenicity events in the 6-day or 2-day post-vaccination period for either the booster or primary series doses……………………………………………………………………………………………………….4
Table S2. Healthcare resource utilization among booster dose recipients over the 6-day post-vaccination period……...5
Figure S1. Baseline survey…………………………………………………………………………………………………6
Figure S2. Standardized mean differences plot for the 6-day post-vaccination booster sample…………………………..26
Figure S3. Propensity score distribution for the 6-day post-vaccination booster sample…………………………………..27
Figure S4. Responses to Vaccine Symptoms diary (reactogenicity) for the 2-day post-vaccination period for the Booster Dose and Primary Series Populations…………………………………………………………………………………...28
References………..29

Supplemental Methods

Propensity score development/assignment procedure:

The comparative analyses were weighted using stabilized inverse probability of treatment weights (sIPTW) [1,2]. These weights were developed independently for the day 6 post-vaccination period and the days 1-2 post-vaccination period, for the Booster Dose and Primary Series Populations (example shown in **Figures S2-3** for booster at day 6).

For categorical endpoints, comparative analyses were conducted using a sIPTW-weighted logistic regression model. For continuous endpoints, comparative analyses were conducted using an sIPTW-weighted analysis of variance (ANOVA) model. All data analyses were performed using SAS statistical software, version 9.4 (SAS; Cary, NC). The following steps were used to develop each set of propensity scores:

1) Identify covariates potentially associated with the outcome of interest from among the relevant demographic and clinical characteristics collected in the questionnaires.
2) Summarize the distributions of the covariates derived in step 1 within the NVX-CoV2373 and mRNA vaccine groups and assess them for similarity using standardized differences [3].
3) Fit a multivariate logistic regression model with vaccine group as the dependent variable and all of the covariates identified in step 1 as independent variables to create a propensity score for each participant.
4) Assess the magnitude of the overlap between the propensity score distributions for the NVX-CoV2373 and mRNA vaccine groups using histograms of the propensity score distributions for each group.
5) For each participant, calculate the sIPTW for the propensity score by multiplying the inverse of the participant's modeled propensity score by the proportion of participants who received the vaccine that the participant received.
6) Check again for similarity of the covariate distributions within the NVX-CoV2373 and mRNA vaccine groups using standardized differences after the participants in each group are weighted by the propensity scores calculated in step 5. If distributional similarity is still lacking for some covariates, refine the logistic regression model by including interaction terms or higher order terms and repeat the process described in steps 3 through 6 [4,5].

For step 1, the following covariates were used in the propensity score model:

- Booster dose 6-day postvaccination analysis sample: Country, Prior COVID-19 diagnosis, Race (Asian, Hispanic, White), Job Category (Professional, Prefer not to answer), Essential worker status (Prefer not to answer), Gender identity (Male, Female), Scheduled to work in the next 24 hours.
- Booster dose days 1 and 2 postvaccination analysis sample: Country, Prior COVID-19 diagnosis, Race (Asian, White), Job Category (Professional), Work at home, Gender identity (Male, Female), Scheduled to work in the next 24 hours.
- Primary series 6-day postvaccination analysis sample: Country, Age, Prior COVID-19 diagnosis, Any high risk medical conditions, Race (White), Gender identity (Male, Female), Job category (Sales, Precision production or craft worker), Scheduled to work in the next 24 hours.
- Primary series days 1 and 2 postvaccination analysis sample: Age, Scheduled to work in the next 24 hours, Gender identity (Male, Female), Race (White).

For step 3, the following multivariate logistic regression model was used to model the log odds of receiving the mRNA vaccine conditional on the covariates listed above as independent variables to obtain the predicted probability of being assigned to the mRNA vaccine group for each participant:

$$log\left(\frac{p_{mRNA}}{1-p_{mRNA}}\right) = \alpha + \beta_1 x_1 + \beta_2 x_2 + \cdots + \beta_n x_n$$

where p_{mRNA} is the probability of receiving the mRNA vaccine, α is the intercept, and β_1, β_2, ..., β_n are the regression coefficients for the covariates x_1, x_2, ..., x_n listed above.

Solving for p_{mRNA}, the propensity score (PS) is obtained by:

$$PS = p_{mRNA} = \frac{exp\{\alpha+\beta_1 x_1+\beta_2 x_2+\cdots+\beta_n x_n\}}{1+exp\{\alpha+\beta_1 x_1+\beta_2 x_2+\cdots+\beta_n x_n\}} \quad \text{(Equation 1).}$$

At this point (step 4), the magnitude of the overlap between the *PS* distributions for the NVX-CoV2373 and mRNA vaccine groups was assessed. This was done by examining histograms of the *PS* distributions for each group. Once satisfactory overlap was achieved, propensity score weights equal to the stabilized inverse of the probability of the vaccine received were derived.

For step 5, the stabilized propensity score weight will be derived. First, the proportion of participants receiving each vaccine will be determined ($Prop_{mRNA}$ and $1 - Prop_{mRNA}$). Using this information and the propensity score obtained in Equation 1, the following stabilized propensity score weight will be assigned to each mRNA participant:

$$Weight_{mRNA} = \frac{Prop_{mRNA}}{PS} 1 - Prop_{mRNA}$$

And the following stabilized propensity score weight will be assigned to each NVX-CoV2373 participant:

$$Weight_{NVX} = \frac{1 - Prop_{mRNA}}{1 - PS}$$

The above weights will be used to adjust the comparative analyses between vaccine groups to reduce the bias that may have existed due to differences in the underlying characteristics of participants in the 2 vaccine groups.

Table S1. Percentage of any reactogenicity events in the 6-day or 2-day post-vaccination period for either the booster or primary series doses.

Parameter, %	Booster Dose Population		Primary Series Population	
	NVX-CoV2373 (n = 303)	mRNA vaccine (n = 827)	NVX-CoV2373 (n = 135)	mRNA vaccine (n = 102)
**6-day post-vaccination period**				
**Local reactogenicity events**				
Injection site pain	55.8	86.2	45.9	73.5
Injection site tenderness	63.7	89.1	56.3	75.5
Injection site erythema	16.5	29.5	15.6	26.5
Injection site swelling	20.5	42.9	17.8	36.3
**Systemic reactogenicity events**				
Elevated temperature	8.6	20.6	7.4	15.7
Fatigue	45.9	71.6	34.8	47.1
Malaise	37.6	62.5	34.1	44.1
Muscle pain	43.9	74.7	42.2	61.8
Joint pain	30.0	47.5	27.4	38.2
Nausea or vomiting	14.2	17.7	14.1	14.7
Headache	35.3	53.6	28.9	33.3
**2-day post-vaccination period**				
**Local reactogenicity events**				
Injection site pain	54.8	85.8	45.7	72.4
Injection site tenderness	63.3	89.1	55.8	76.5
Injection site erythema	13.9	27.6	13.2	24.5
Injection site swelling	19.7	41.6	15.5	35.7
**Systemic reactogenicity events**				
Elevated temperature	6.5	18.0	7.0	14.3
Fatigue	42.5	68.1	32.6	41.8
Malaise	31.3	58.7	32.6	38.8
Muscle pain	40.1	73.2	39.5	59.2
Joint pain	24.1	44.5	24.8	34.7
Nausea or vomiting	8.5	13.4	10.1	12.2
Headache	29.3	48.4	27.9	28.6

Table S2. Healthcare resource utilization among booster dose recipients over the 6-day post-vaccination period.

**Parameter**	**NVX-CoV2373 (n = 303)**	**mRNA vaccine (n = 827)**
Use of over-the-counter medications, n (%)	52 (18.5%)	178 (22.7%)
Use of prescription medications, n (%)	2 (0.7)	16 (2.0)
Adjusted use of over-the-counter or prescription medications, % (95% CI)	18.8% (14.2, 23.4)	22.8% (19.6, 25.9)
Doctor's office or telemedicine visit, n (%)	3 (1.1)	8 (1.0)
Urgent care center visits, n (%)	1 (0.4)	4 (0.5)
Emergency department visits, n (%)	1 (0.4)	2 (0.3)
Overnight hospitalization, n (%)	3 (1.1)	2 (0.3)
Intensive care unit admission, n (%)	1 (0.4)	1 (0.1)

Figure S1. Baseline survey.

Burden and Impact of Reactogenicity Among US Adults Receiving COVID-19 Vaccines

(VIP Survey)

Baseline Questionnaire (Day 0)

This survey is about your experiences with receiving a COVID-19 vaccination. You will be asked to complete a short survey each day for 6 days, starting tomorrow. For today, we'd like to ask a few questions about your prior experience with COVID-19 and COVID-19 vaccination. Then we'll ask a few questions about yourself.

B1. Have you ever been diagnosed with COVID-19 (received a positive test or been told by a healthcare provider)?

☐ Yes

☐ No

B2. Have you been told you have a medical condition that puts you at high risk for a severe COVID-19 infection?

☐ Yes

☐ No

B2a. Please select the medical conditions that put you at high risk for severe COVID-19 infection. (*Please select all that apply*.)

☐ Diabetes

☐ Hypertension

☐ Heart disease

☐ Respiratory conditions

☐ Other

B3. Were you required to get a COVID-19 vaccine as part of a vaccine requirement (for example, as required by your employer)?

- ☐ Yes
- ☐ No

B4. Did you have a choice of more than one COVID-19 vaccine brand today?

- ☐ Yes
- ☐ No

B4a. Did you get the vaccine brand you wanted?

- ☐ Yes
- ☐ No

B5. Are you scheduled to work in the next 24 hours?

- ☐ Yes
- ☐ No

B5a. Did you intentionally schedule your vaccine for a time when you knew you would not have to work the next day?

- ☐ Yes
- ☐ No

B5b. Did you purposely change your work schedule to take the next day off after receiving your COVID-19 vaccine?

- ☐ Yes
- ☐ No

The next few questions are about yourself.

B6. What gender identity best describes you? *(Please select one.)*

- ☐ Female
- ☐ Male
- ☐ Gender fluid
- ☐ Nonbinary
- ☐ A gender identity not listed
- ☐ Prefer not to answer

B7. What race(s) or ethnicity(ies) do you consider yourself to be? (*Please select all that apply*.)

- ☐ African American or Black
- ☐ Alaska Native, American Indian, Native American
- ☐ Asian
- ☐ Hispanic, Latin American, or Latinx
- ☐ Middle Eastern or North African
- ☐ Native Hawaiian or Pacific Islander
- ☐ White
- ☐ A race or ethnicity not listed
- ☐ Prefer not to answer

B8. What is the highest grade or level of school that you have completed? (*Please choose one answer only.*)

- ☐ Less than high school
- ☐ High school diploma or equivalent (for example, GED)
- ☐ Some college
- ☐ College degree
- ☐ Professional or advanced degree
- ☐ Prefer not to answer

B9. Do you currently work from home or work outside of the home <u>most</u> days?

- ☐ Work from home
- ☐ Work outside the home
- ☐ Prefer not to answer

B10. Please choose the category that best describes your main job. If none of the categories fits you exactly, please respond with the closest category.

- ☐ Executive, administrator, or senior manager (for example, chief executive officer, sales vice president, plant manager)
- ☐ Professional (for example, engineer, accountant, systems analyst)
- ☐ Technical support (for example, lab technician, legal assistant, computer programmer)
- ☐ Sales (for example, sales representative, stockbroker, retail sales)
- ☐ Clerical and administrative support (for example, secretary, billing clerk, office supervisor)
- ☐ Service occupation (for example, security officer, food service worker, janitor)
- ☐ Precision production and crafts worker (for example, mechanic, carpenter, machinist)

- ☐ Operator or laborer (for example, assembly line worker, truck driver, construction worker)
- ☐ Active member of US military
- ☐ Prefer not to answer

B11. What is your annual income from all employment before taxes?

- ☐ $1 to $9,999
- ☐ $10,000 to $24,999
- ☐ $25,000 to $49,999
- ☐ $50,000 to $99,999
- ☐ $100,000 to $199,999
- ☐ $200,000 to $499,999
- ☐ $500,000 or more
- ☐ Prefer not to answer

B12. Are you considered an essential worker?

- ☐ Yes
- ☐ No
- ☐ Prefer not to answer

Thank you for completing this baseline survey! You will receive a $25 electronic pre-paid card for completing this survey today. As part of this survey, you are being asked to complete short surveys daily for the next 6 days. You will receive a reminder/invitation tomorrow around 4 pm to complete tomorrow's survey. You will have until midnight to complete the survey. After midnight, the survey will be closed for that day. Please try to complete the survey at approximately the same time each day. This should be around the end of your normally scheduled workday. If you work different shifts during the

course of the survey then please try to complete the survey by midnight (12 am). You can collect one $25 electronic pre-paid card for each of the days you complete a survey.

Thank you again for participating. Once you have read this screen, please hand the tablet to the site coordinator.

Day 1 Diary

Good evening! Your daily survey is available for completion. Please complete this survey by midnight tonight in order to receive a $25 electronic pre-paid card. You should complete the survey at the end of your normally scheduled workday or at the end of your shift, but before midnight. Please try to complete the survey at approximately the same time each day.

The first few questions are about your experiences yesterday **AFTER** receiving your COVID-19 vaccination. This means you should answer these questions thinking **only** about the time from when you received your vaccine until midnight last night.

1. Were you scheduled or had you planned to work yesterday AFTER receiving your COVID-19 vaccination?

- ☐ Yes
- ☐ No

2. How many hours were you scheduled or had you planned to work yesterday AFTER receiving your COVID-19 vaccination?

______ HOURS

3. After receiving your vaccination yesterday, did you miss any of your planned/scheduled work because of vaccination-related symptoms? Do not include time you missed to participate in this survey or to get your COVID-19 vaccine.

- ☐ Yes

☐ No

4. How many hours did you miss from work yesterday AFTER receiving your vaccination because of problems associated with your COVID-19 vaccination? Include hours you missed due to feeling sick, going in late, leaving early, etc., because of your COVID-19 vaccination-related symptoms. Do not include time you missed to participate in this survey or to get this COVID-19 vaccine.

_____ HOURS

5. How much did your COVID-19 vaccination affect your productivity while you were working yesterday AFTER receiving your vaccination? Think about the hours you were limited in the amount or kind of work you could do, hours you accomplished less than you would like, or hours you could not do your work as carefully as usual.

If your COVID-19 vaccination affected your work only a little, choose a low number. Choose a high number if your COVID-19 vaccination affected your work a great deal. Consider only how much your COVID-19 vaccination affected productivity while you were working.

My COVID-19 vaccination had no effect on my work	0	1	2	3	4	5	6	7	8	9	10	My COVID-19 vaccination completely prevented me from working

6. How much did your COVID-19 vaccination affect your ability to do your regular daily activities (other than work at a job) yesterday AFTER receiving your vaccination? By regular activities, we mean the usual activities you do, such as work around the house, shopping, childcare, exercising, studying, etc. Think about time you were limited in the amount or kind of activities you could do and time you accomplished less than you would like.

If your COVID-19 vaccination affected your activities only a little, choose a low number. Choose a high number if your COVID-19 vaccination affected your activities a great deal. Consider only how much your COVID-19 vaccination affected your ability to do your regular daily activities, other than work at a job.

My COVID-19 vaccination had no effect on my daily activities	0	1	2	3	4	5	6	7	8	9	10	My COVID-19 vaccination completely prevented me from doing my daily activities

The next questions are about your experiences **TODAY**. In answering the questions, please think only about the time since midnight last night until right now.

7. Were you scheduled or had you planned to work today?

☐ Yes

☐ No

8. Thinking about today only, how many hours did you miss from work because of problems associated with your COVID-19 vaccination?

Include hours you missed due to feeling sick, going in late, leaving early, etc., because of your COVID-19 vaccination-related symptoms.

_____ HOURS

9. Thinking about today only, how many hours did you miss from work because of any other reason, such as vacation, holidays, etc.?

_____ HOURS

10. Thinking about today only, how many hours did you actually work?

_____ HOURS

11. **Thinking about today only, how much did your COVID-19 vaccination affect your productivity while you were working?** Think about the hours you were limited in the amount or kind of work you could do, hours you accomplished less than you would like, or hours you could not do your work as carefully as usual.

If your COVID-19 vaccination affected your work only a little, choose a low number. Choose a high number if your COVID-19 vaccination affected your work a great deal. Consider only how much your COVID-19 vaccination affected productivity while you were working.

My COVID-19 vaccination had no effect on my work	0	1	2	3	4	5	6	7	8	9	10	My COVID-19 vaccination completely prevented me from working

SELECT A NUMBER

12. **Thinking about today only, how much did your COVID-19 vaccination affect your ability to do your regular daily activities, other than work at a job?** By regular activities, we mean the usual activities you do, such as work around the house, shopping, childcare, exercising, studying, etc. Think about time you were limited in the amount or kind of activities you could do and time you accomplished less than you would like.

If your COVID-19 vaccination affected your activities only a little, choose a low number. Choose a high number if your COVID-19 vaccination affected your activities a great deal. Consider only how much your COVID-19 vaccination affected your ability to do your regular daily activities, other than work at a job.

My COVID-19 vaccination had no effect on my daily activities	0	1	2	3	4	5	6	7	8	9	10	My COVID-19 vaccination completely prevented me from doing my daily activities

SELECT A NUMBER

The next questions are about possible side effects you may experience from the COVID-19 vaccine. Please think about the maximum severity of the specific vaccine symptom you have experienced today.

Do your best to assign a value from 0 to 3. Please look at the descriptions beside each number to choose the value that best fits your symptom.

13. Injection site pain – Record maximum pain experienced today

- ☐ 0 – No pain
- ☐ 1 – No interference with activity
- ☐ 2 – Repeated use of nonprescription pain reliever **OR** interferes with activity
- ☐ 3 – Any use of prescription pain reliever **OR** prevents daily activity

14. Injection site tenderness – Record maximum tenderness experienced today

- ☐ 0 – No tenderness
- ☐ 1 – Mild discomfort to touch
- ☐ 2 – Discomfort with movement
- ☐ 3 – Significant discomfort at rest

15. Injection site redness – Record maximum redness experienced today

- ☐ 0 – No redness
- ☐ 1 – Mild redness
- ☐ 2 – Moderate redness
- ☐ 3 – Significant redness

16. Injection site swelling – Record maximum swelling experienced today

- ☐ 0 – No swelling
- ☐ 1 – Mild swelling
- ☐ 2 – Moderate swelling
- ☐ 3 – Significant swelling

17. Fever measured or not measured (use maximum recorded temperature, if available) – Answer based on the maximum fever today

☐ 0 – No fever (< 100.4 °F) (< 38.0 °C)

☐ 1 – Mild fever (100.4 to 101.1 °F) (38.0 to 38.4 °C)

☐ 2 – Moderate fever (101.2 to 102.0 °F) (38.5 to 38.9 °C)

☐ 3 – Significant fever (> 102.0 °F) (> 38.9 °C)

18. Fatigue – Record maximum intensity experienced today

☐ 0 – Not present

☐ 1 – No interference with activity

☐ 2 – Some interference with activity

☐ 3 – Significant interference with activity

19. Malaise/feeling sick (general feeling of discomfort or unease excluding "fatigue, muscle pain, or joint pain") – Record maximum intensity experienced today

☐ 0 – Not present

☐ 1 – No interference with activity

☐ 2 – Some interference with activity

☐ 3 – Significant interference with activity

20. Muscle pain – Record maximum intensity experienced today

☐ 0 – Not present

☐ 1 – No interference with activity

☐ 2 – Some interference with activity

☐ 3 – Significant interference with activity

21. Joint pain – Record maximum intensity experienced today

- ☐ 0 – Not present
- ☐ 1 – No interference with activity
- ☐ 2 – Some interference with activity
- ☐ 3 – Significant interference with activity

22. Nausea/vomiting – Record nausea/vomiting experienced today

- ☐ 0 – No nausea/vomiting
- ☐ 1 – Mild, no interference with activity or 1-2 episodes today
- ☐ 2 – Moderate, some interference with activity or > 2 episodes today
- ☐ 3 – Severe, prevents daily activity or requires intravenous hydration outside of hospital

23. Headache – Record maximum headache experienced today

- ☐ 0 – No pain
- ☐ 1 – No interference with activity
- ☐ 2 – Repeated use of nonprescription pain reliever **OR** interferes with activity
- ☐ 3 – Any use of prescription pain reliever **OR** prevents daily activity

Thank you for completing this survey! You will be sent an email with a $25 electronic pre-paid card. You will receive a reminder/invitation tomorrow around 4 pm to complete tomorrow's survey. You will have until midnight to complete the survey. Please try to complete the survey at approximately the same time each day. This should be around the end of your normally scheduled workday. If you work different shifts during the course of the survey, then please try to complete the survey by midnight (12 am).

Day 2-6 Diary

Good evening! Your daily survey is available for completion. Please complete this survey by midnight tonight in order to receive a $25 electronic pre-paid card. Please try to complete the survey at approximately the same time as you did yesterday. This should be around the end of your normally scheduled workday or at the end of your shift.

The following questions are about your experiences in the past 24 hours.

1. Were you scheduled or had you planned to work?

☐ Yes

☐ No

2. During the past 24 hours, how many hours did you miss from work because of problems associated with your COVID-19 vaccination?

Include hours you missed due to feeling sick, going in late, leaving early, etc., because of your COVID-19 vaccination-related symptoms.

______ HOURS

3. During the past 24 hours, how many hours did you miss from work because of any other reason, such as vacation, holidays, etc.?

______ HOURS

4. During the past 24 hours, how many hours did you actually work?

______ HOURS

5. **During the past 24 hours, how much did your COVID-19 vaccination affect your productivity while you were working?** Think about the hours you were limited in the amount or kind of work you could do, hours you accomplished less than you would like, or hours you could not do your work as carefully as usual.

If your COVID-19 vaccination affected your work only a little, choose a low number. Choose a high number if your COVID-19 vaccination affected your work a great deal. Consider only how much your COVID-19 vaccination affected productivity while you were working.

My COVID-19 vaccination had no effect on my work	0	1	2	3	4	5	6	7	8	9	10	My COVID-19 vaccination completely prevented me from working

SELECT A NUMBER

6. **During the past 24 hours, how much did your COVID-19 vaccination affect your ability to do your regular daily activities, other than work at a job?** By regular activities, we mean the usual activities you do, such as work around the house, shopping, childcare, exercising, studying, etc. Think about time you were limited in the amount or kind of activities you could do and time you accomplished less than you would like.

If your COVID-19 vaccination affected your activities only a little, choose a low number. Choose a high number if your COVID-19 vaccination affected your activities a great deal. Consider only how much your COVID-19 vaccination affected your ability to do your regular daily activities, other than work at a job.

My COVID-19 vaccination had no effect on my daily activities	0	1	2	3	4	5	6	7	8	9	10	My COVID-19 vaccination completely prevented me from doing my daily activities

SELECT A NUMBER

The next questions are about possible side effects you may experience from the COVID-19 vaccine. Please think about the maximum severity of the specific vaccine symptom you have experienced **during the past 24 hours**. Do your best to assign a value from 0 to 3. Please look at the descriptions under each number to choose the value that best fits your symptom.

7. Injection site pain – Record maximum pain experienced in the past 24 hours

- ☐ 0 – No pain
- ☐ 1 – No interference with activity
- ☐ 2 – Repeated use of nonprescription pain reliever **OR** interferes with activity
- ☐ 3 – Any use of prescription pain reliever **OR** prevents daily activity

8. Injection site tenderness – Record maximum tenderness experienced in the past 24 hours

- ☐ 0 – No tenderness
- ☐ 1 – Mild discomfort to touch
- ☐ 2 – Discomfort with movement
- ☐ 3 – Significant discomfort at rest

9. Injection site redness – Record maximum redness experienced in the past 24 hours

- ☐ 0 – No redness
- ☐ 1 – Mild redness
- ☐ 2 – Moderate redness
- ☐ 3 – Significant redness

10. Injection site swelling – Record maximum swelling experienced in the past 24 hours

- ☐ 0 – No swelling
- ☐ 1 – Mild swelling
- ☐ 2 – Moderate swelling

☐ 3 – Significant swelling

11. Fever measured or not measured (use maximum recorded temperature, if available) – Answer based on the maximum fever in the past 24 hours

☐ 0 – No fever (< 100.4 °F) (< 38.0 °C)

☐ 1 – Mild fever (100.4 to 101.1 °F) (38.0 to 38.4 °C)

☐ 2 – Moderate fever (101.2 to 102.0 °F) (38.5 to 38.9 °C)

☐ 3 – Significant fever (> 102.0 °F) (> 38.9 °C)

12. Fatigue – Record maximum intensity experienced in the past 24 hours

☐ 0 – Not present

☐ 1 – No interference with activity

☐ 2 – Some interference with activity

☐ 3 – Significant interference with activity

13. Malaise/feeling sick (general feeling of discomfort or unease excluding "fatigue, muscle pain, or joint pain") – Record maximum intensity experienced in the past 24 hours

☐ 0 – Not present

☐ 1 – No interference with activity

☐ 2 – Some interference with activity

☐ 3 – Significant interference with activity

14. Muscle pain – Record maximum intensity experienced in the past 24 hours

☐ 0 – Not present

☐ 1 – No interference with activity

☐ 2 – Some interference with activity

☐ 3 – Significant interference with activity

15. Joint pain – Record maximum intensity experienced in the past 24 hours

☐ 0 – Not present

☐ 1 – No interference with activity

☐ 2 – Some interference with activity

☐ 3 – Significant interference with activity

16. Nausea/vomiting – Record nausea/vomiting experienced in the past 24 hours

☐ 0 – No nausea/vomiting

☐ 1 – Mild, no interference with activity or 1-2 episodes/24 hours

☐ 2 – Moderate, some interference with activity or > 2 episodes/24 hours

☐ 3 – Severe, prevents daily activity or requires intravenous hydration outside of hospital

17. Headache – Record maximum headache experienced in the past 24 hours

☐ 0 – No pain

☐ 1 – No interference with activity

☐ 2 – Repeated use of nonprescription pain reliever **OR** interferes with activity

☐ 3 – Any use of prescription pain reliever **OR** prevents daily activity

Thank you for completing this survey! You will be sent an email with a $25 electronic pre-paid card. You will receive a reminder/invitation tomorrow around 4 pm to complete tomorrow's survey. You will have until midnight to complete the survey. Please try to complete the survey at approximately the same time each day. This should be around the end of your normally scheduled workday. If you work different shifts during the course of the survey then please try to complete the survey by midnight (12 am).

End of Survey Healthcare Resource Use Questions (Day 6)

For this last set of questions, think about the last 7 days, that is, since you received your COVID-19 vaccine.

18. Did you use any over-the-counter medications because of symptoms related to your COVID-19 vaccine?

☐ Yes

☐ No

How many days? ____________

19. Did you require prescription medications because of symptoms related to your COVID-19 vaccine?

☐ Yes

☐ No

How many days? ____________

20. Did you visit your doctor's office, or have a telemedicine visit because of symptoms related to your COVID-19 vaccine?

☐ Yes

☐ No

How many visits? ____________

21. Did you visit an urgent care center because of symptoms related to your COVID-19 vaccine?

☐ Yes

☐ No

How many visits? ____________

22. Did you visit a hospital emergency department because of symptoms related to your COVID-19 vaccine?

☐ Yes

☐ No

How many visits? ____________

23. Were you hospitalized overnight because of symptoms related to your COVID-19 vaccine?

☐ Yes

☐ No

How many nights? ____________

Please check the vaccine-related side effects related to hospital admission: (***Please select all that apply.***)

- ☐ Injection site pain
- ☐ Injection site tenderness
- ☐ Injection site swelling
- ☐ Injection site redness
- ☐ Fever
- ☐ Fatigue
- ☐ Malaise
- ☐ Muscle pain
- ☐ Joint pain
- ☐ Nausea/vomiting
- ☐ Headache
- ☐ Other

24. Were you admitted to an intensive care unit (ICU) because of symptoms related to your COVID-19 vaccine?

☐ Yes

☐ No

How many nights? ____________

Please check the vaccine-related side effects related to ICU admission: **(*Please select all that apply*.)**

☐ Injection site pain

☐ Injection site tenderness

☐ Injection site swelling

☐ Injection site redness

☐ Fever

☐ Fatigue

☐ Malaise

☐ Muscle pain

☐ Joint pain

☐ Nausea/vomiting

☐ Headache

☐ Other

Thank you for completing this survey! You will be sent an email with a $25 electronic pre-paid card. This was your final survey to complete as part of the survey. If you plan on receiving another COVID-19 vaccine in the future, you may qualify to repeat the survey just as you have for the last 7 days. If you have any questions about your participation in this research, please contact the survey team at VIPSurvey@rti.org.

We sincerely thank you for participating in this important survey!

Figure S2: Standardized mean differences plot for the 6-day post-vaccination booster sample.

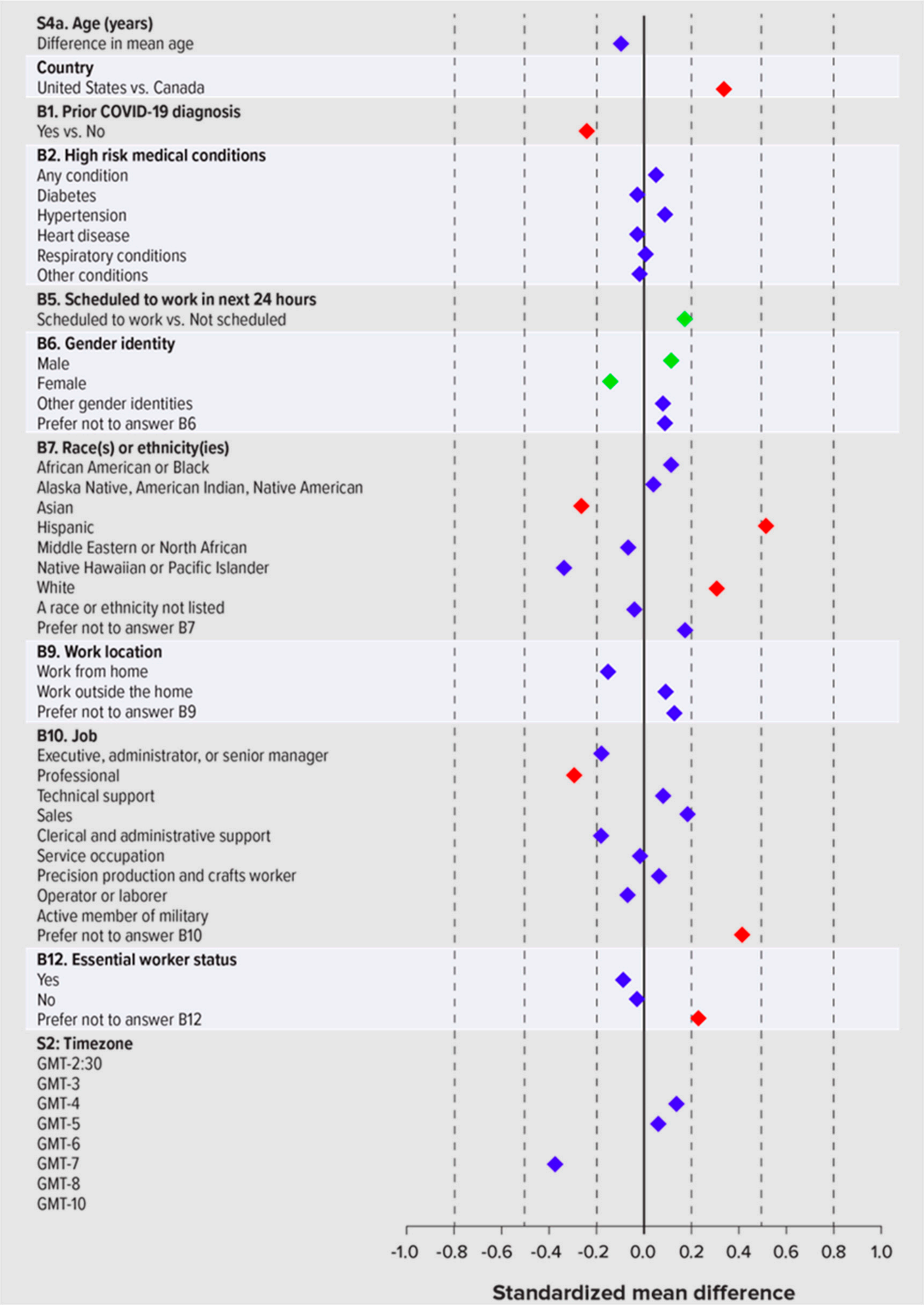

GMT = Greenwich Mean Time; SMD = standardized mean difference.

Note: Red shaded covariates included in the propensity score model based on an |SMD| > 0.2. Green shaded covariates were forced into the propensity score model based on their clinical relevance.

Figure S3: Propensity score distribution for the 6-day post-vaccination booster sample.

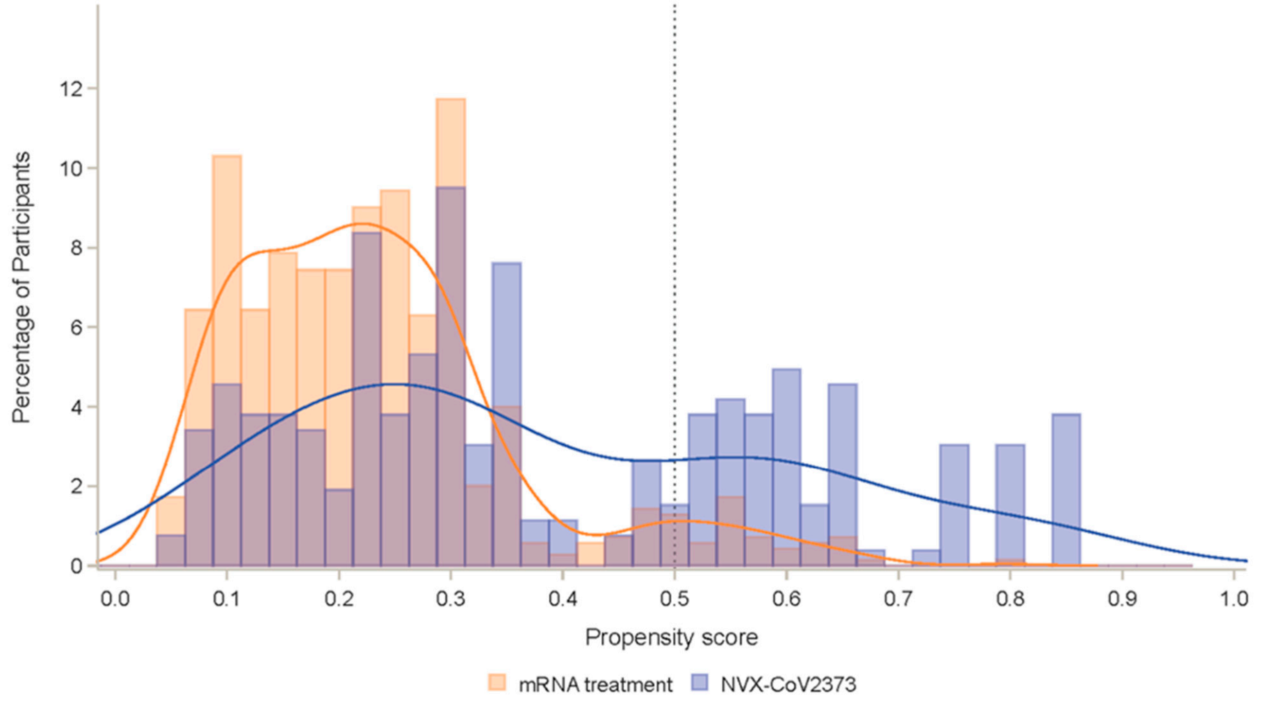

mRNA = messenger RNA.

Figure S4. Responses to Vaccine Symptoms diary (A, local, and B, systemic reactogenicity) at days 1 and 2 post-vaccination for the Booster Dose and Primary Series Populations.

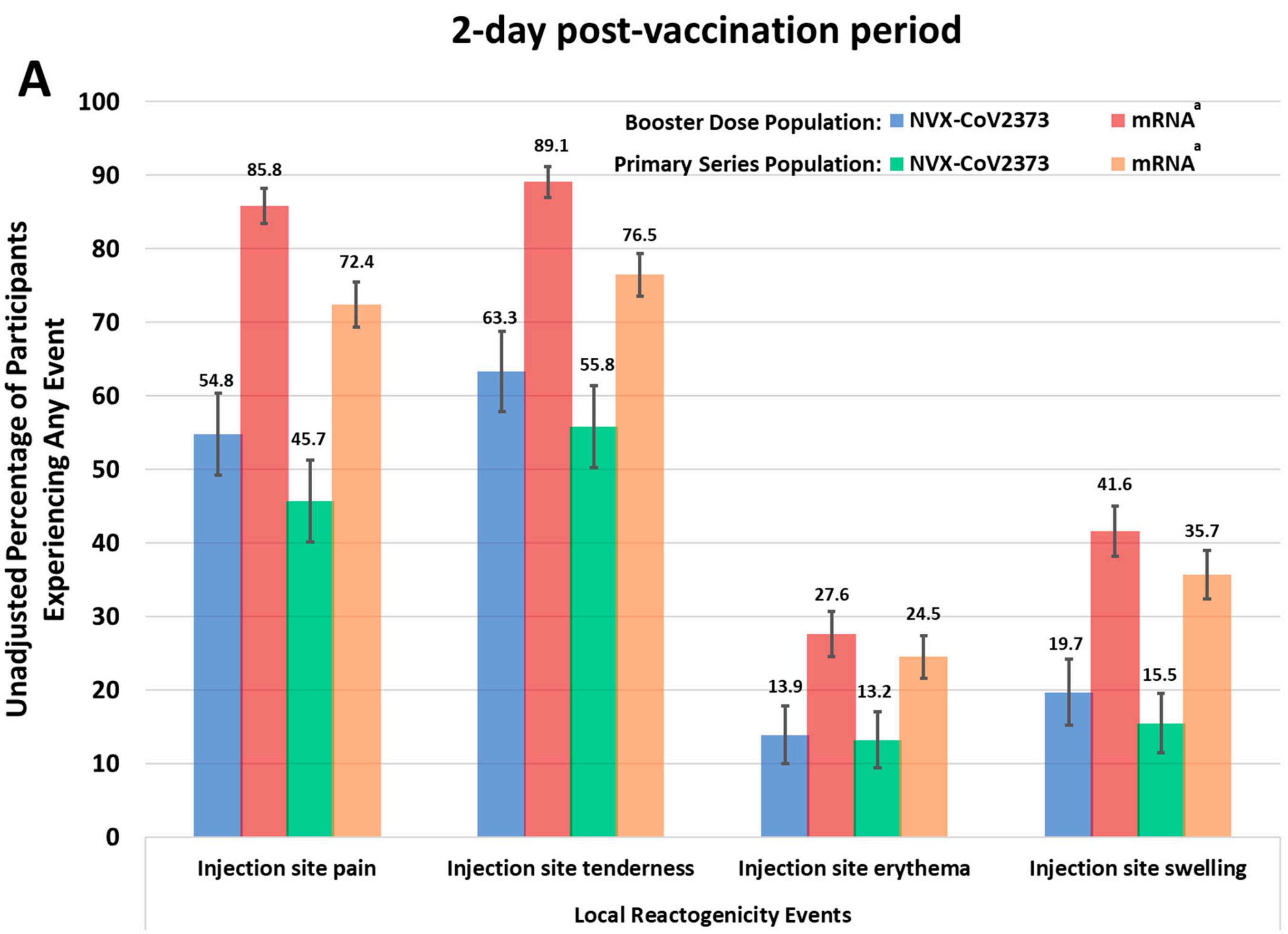

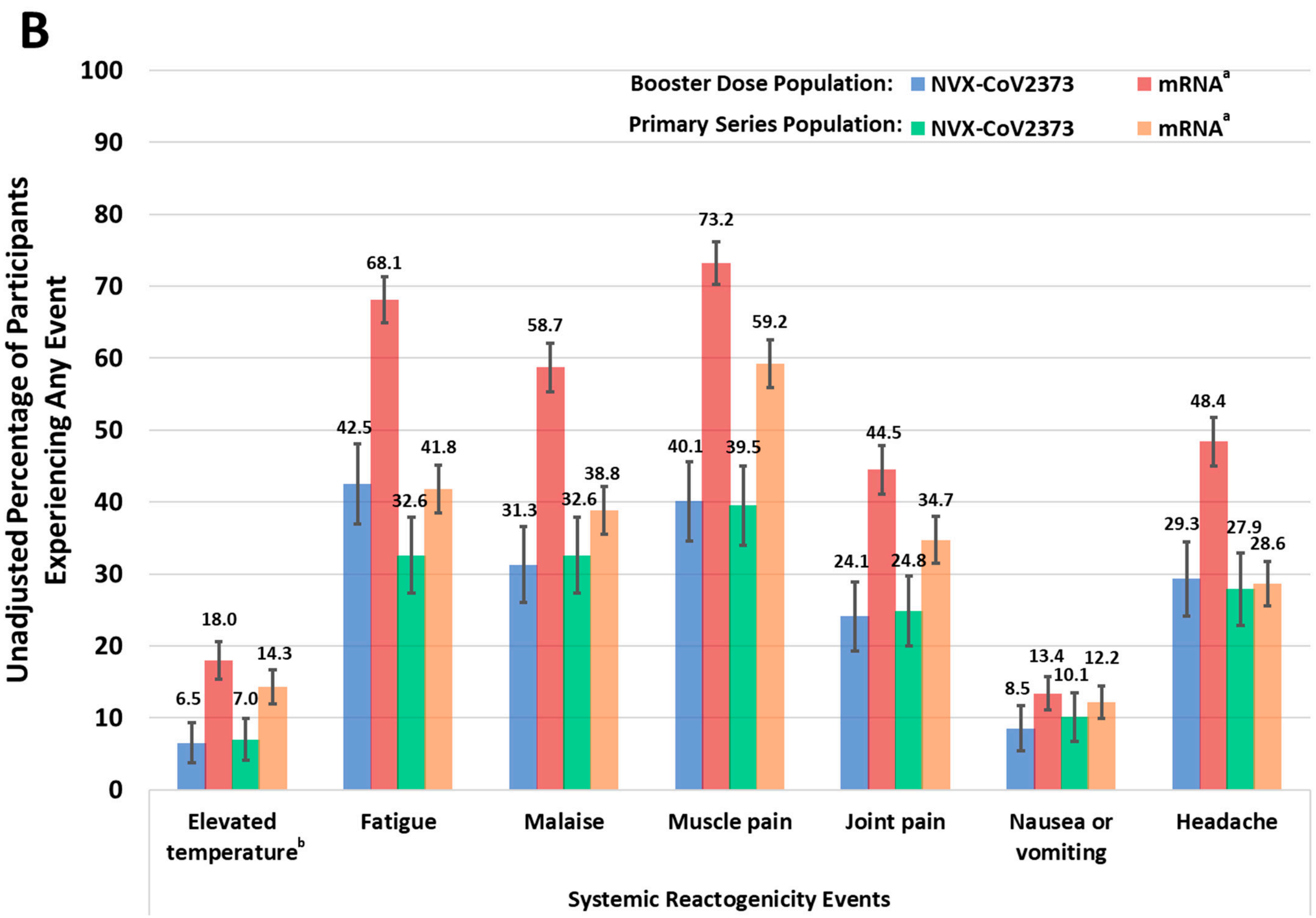

Participants experienced an event if a grade 1 o worse reactogenicity event was reported for any day during the 2-day post-vaccination period.
[a] Individuals received either BNT162b2 or mRNA-1273.
[b] Temperatures at or above 100.4°F were considered elevated.

References:

1. D'Agostino RB Jr. Propensity score methods for bias reduction in the comparison of a treatment to a non-randomized control group. Stat Med. 1998;17(19):2265-81.
2. Robins JM, Hernan MA, Brumback B. Marginal structural models and causal inference in epidemiology. Epidemiology 2000 Sep;11(5):550-56.
3. Austin, P.C. Balance diagnostics for comparing the distribution of baseline covariates between treatment groups in propensity-score matched samples. *Stat Med* **2009**, *28*, 3083-3107, doi:10.1002/sim.3697.
4. Glynn R, Schneeweiss S, Stürmer T. Indications for propensity scores and review of their use in pharmacoepidemiology. Basic Clin Pharmacol Toxicol. 2006;98(3):253-9. doi: 10.1111/j.1742-7843.2006.pto_293.x. PMID: 16611199; PMCID: PMC1790968.
5. Perkins SM, Tu W, Underhill MG, Zhou XH, Murray MD. The use of propensity scores in pharmacoepidemiologic research. Pharmacoepidemiol Drug Saf. 2000;9(2):93-101.
